# The Multiples Fates of the Flavivirus RNA Genome During Pathogenesis

**DOI:** 10.3389/fgene.2018.00595

**Published:** 2018-12-04

**Authors:** Clément Mazeaud, Wesley Freppel, Laurent Chatel-Chaix

**Affiliations:** Institut National de la Recherche Scientifique, Centre INRS-Institut Armand-Frappier, Laval, QC, Canada

**Keywords:** flavivirus, dengue virus, Zika virus, West Nile virus, viral RNA replication, translation, RNA encapsidation, innate immunity

## Abstract

The *Flavivirus* genus comprises many viruses (including dengue, Zika, West Nile and yellow fever viruses) which constitute important public health concerns worldwide. For several of these pathogens, neither antivirals nor vaccines are currently available. In addition to this unmet medical need, flaviviruses are of particular interest since they constitute an excellent model for the study of spatiotemporal regulation of RNA metabolism. Indeed, with no DNA intermediate or nuclear step, the flaviviral life cycle entirely relies on the cytoplasmic fate of a single RNA species, namely the genomic viral RNA (vRNA) which contains all the genetic information necessary for optimal viral replication. From a single open reading frame, the vRNA encodes a polyprotein which is processed to generate the mature viral proteins. In addition to coding for the viral polyprotein, the vRNA serves as a template for RNA synthesis and is also selectively packaged into newly assembled viral particles. Notably, vRNA translation, replication and encapsidation must be tightly coordinated in time and space via a fine-tuned equilibrium as these processes cannot occur simultaneously and hence, are mutually exclusive. As such, these dynamic processes involve several vRNA secondary and tertiary structures as well as RNA modifications. Finally, the vRNA can be detected as a foreign molecule by cytosolic sensors which trigger upon activation antiviral signaling pathways and the production of antiviral factors such as interferons and interferon-stimulated genes. However, to create an environment favorable to infection, flaviviruses have evolved mechanisms to dampen these antiviral processes, notably through the production of a specific vRNA degradation product termed subgenomic flavivirus RNA (sfRNA). In this review, we discuss the current understanding of the fates of flavivirus vRNA and how this is regulated at the molecular level to achieve an optimal replication within infected cells.

## Introduction

Infections with flaviviruses constitute a major public health concern worldwide since they cause several human diseases with a wide range of symptoms that can potentially lead to lifelong impairment or even death. The genus *Flavivirus* within the *Flaviviridae* virus family comprises almost 70 reported species including the most studied yellow fever virus (YFV), dengue virus (DENV), Zika virus (ZIKV), West Nile virus (WNV), Japanese encephalitis virus (JEV), and tick-borne encephalitis virus (TBEV). The vast majority of flaviviral infections in humans occur through the biting by arthropods such as *Aedes*-type mosquitoes (mostly *Aedes aegypti* and *Aedes albopictus*) in the case of YFV, DENV, and ZIKV or *Culex pipiens* mosquitoes in the case of WNV. Vaccines do exist for YFV, DENV and TBEV. However, in the case of DENV, the cause of the most prevalent arthropod-borne viral disease, the only available vaccine shows limited efficacy against all DENV serotypes and safety concerns have recently arisen in the Philippines in vaccinated children (Dyer, 2017). Importantly, no antivirals against flaviviruses are currently available partly because of our limited understanding of their life cycle and pathogenesis when compared to other virus groups. Interestingly, it appears that the general features of the life cycle are conserved across flaviviruses. Hence, there have been tremendous efforts by both industry and academia to identify or engineer antiviral drugs with a panflaviviral spectrum. This illustrates the importance of deciphering the molecular mechanisms underlying the flavivirus life cycle in order to identify novel antiviral targets.

The flavivirus life cycle is completely dependent on the cytoplasmic fate of only one RNA species, namely the genomic viral RNA (vRNA) whose replication entirely occurs in the cytoplasm and does not generate any DNA intermediates. Most notably, vRNA contains all the genetic information necessary for optimal virus replication. Hence, targeting vRNA or viral processes involved in its metabolism constitutes an attractive strategy for the development of novel antivirals. Moreover, fundamental virology often provides crucial insight into cellular machinery and processes at the molecular level. In this respect, flavivirus vRNA constitutes an exciting and excellent model for investigating the spatiotemporal regulation of RNA metabolism. With that in mind, we focus this review on our current understanding of the multiple fates of vRNA and how it orchestrates the viral life cycle and creates a cellular environment favorable to infection.

**FIGURE 1:**
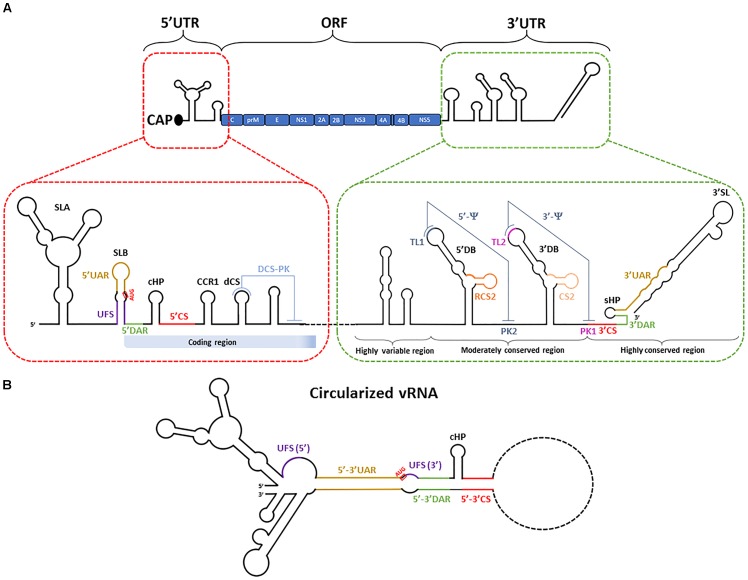
Schematic representation of flavivirus vRNA. **(A)** vRNA is composed of a 5′UTR, one single open reading frame and a 3′UTR. The position of the sequences encoding for the viral proteins within the polyprotein is indicated. The bottom part of the figure shows in details the secondary structures of 5′UTR, capsid-coding region and 3′UTR. The different regions engaged in local pseudoknots and long-range RNA–RNA interactions are indicated and described in detail in the text. **(B)** Predicted structure of vRNA in its circularized conformation. The coding sequence (except 5′ capsid coding region) is depicted with a dashed line.

Flaviviruses are enveloped positive-strand RNA viruses that presumably contain a single copy of the genome RNA. Following receptor-mediated endocytosis of the virion and fusion with the endosomal membrane (reviewed in Perera-Lecoin et al., 2013), the vRNA is uncoated and released into the cytosol. The flaviviral vRNA genome contains all the genetic information required for efficient viral replication by hijacking the intracellular resources. With a single open reading frame, vRNA encodes an endoplasmic reticulum (ER)-associated transmembrane polyprotein (Figure 1A) (Garcia-Blanco et al., 2016; Neufeldt et al., 2018).

Upon translation, the polyprotein is subsequently processed by both cellular and viral proteases to generate 10 mature viral proteins. Structural proteins Capsid (C), Envelop (E) and prM assemble new viral particles while non-structural (NS) proteins NS1, NS2A, NS2B, NS3, NS4A, NS4B, and NS5 are responsible for vRNA replication (Figure 1A). vRNA synthesis relies on NS5, the RNA-dependent RNA polymerase as well as on critical vRNA secondary and tertiary structures. NS5 is also responsible for the capping of the neosynthetized vRNA. NS3 is a protease which, together with its co-factor NS2B, participates to the processing of the viral polyprotein. It also possesses helicase, NTPase and triphosphatase activities, all required for efficient vRNA synthesis and capping. vRNA is then encapsidated into assembling viral particles which bud into the ER. Assembled viruses egress through the secretory pathway where they undergo furin-mediated maturation in the Golgi apparatus, allowing fully infectious virions to be released via exocytosis (Apte-Sengupta et al., 2014; Neufeldt et al., 2018).

In order to efficiently complete the flaviviral life cycle, vRNA translation, synthesis and encapsidation must be tightly coordinated in both time and space since these processes cannot occur simultaneously and hence, are mutually exclusive. However, the molecular mechanisms underlying orchestration of these events remain mostly enigmatic. To achieve such a tight spatiotemporal regulation, flaviviruses, like the vast majority (if not all) of positive-strand RNA viruses, induce massive rearrangements of ER membranes to create a replication-favorable microenvironment that is generically called “replication factories (RF)” (Chatel-Chaix and Bartenschlager, 2014; Paul and Bartenschlager, 2015). These organelle-like ultrastructures host vRNA synthesis among other functions (discussed in more detail below). They are believed to spatially segregate the different steps of the viral life cycle although this model is primarily based on descriptive ultrastructural studies using electron microscopy (Welsch et al., 2009; Gillespie et al., 2010; Miorin et al., 2013; Junjhon et al., 2014; Bily et al., 2015; Cortese et al., 2017). However, there remains a knowledge gap regarding the fate of the vRNA in the cytoplasm of the infected cell. The majority of imaging studies have relied on antibody-based detection of the viral double-stranded (ds) RNA, the positive strand/negative strand hybrid replication intermediate and hence, do not take into consideration vRNA populations engaged in translation or encapsidated into virions. In addition, no detailed fluorescence *in situ* hybridization (FISH)-based sub-cellular distribution analyses of flaviviral vRNA and negative strand intermediate RNA have been reported to date in contrast with those of hepatitis C virus, a non-flavivirus member of the *Flaviviridae* family (Shulla and Randall, 2015).

The 10–11 kb-long flavivirus vRNA genome is composed of one open reading frame (ORF) flanked by highly structured 5′ and 3′ untranslated regions (UTR) (Selisko et al., 2014; Ng et al., 2017). The viral 5′UTR and 3′UTR have been demonstrated to engage in interactions with both host and viral proteins. The 3′UTR can be sub-divided into three sub-domains: (1) a highly variable region located immediately after the stop codon, which is implicated in viral adaptation to the host (Villordo et al., 2015); (2) the moderately conserved region and (3) a highly conserved region (Figure 1A). Most importantly, the vRNA shows a high structural plasticity, as it must undergo conformational changes implicated in the different steps of the viral life cycle. For instance, for efficient genome replication, the genome adopts a “pan-handle”-like circularized structure, which is achieved through long range RNA-RNA interactions between 5′ and 3′ termini. Several circularization motifs (discussed in more detail below) have been identified and are depicted in Figure 1. Interestingly, several sequences and structures in the 5′ and 3′UTRs can harbor multiple functions during distinct steps of the life cycle.

## Viral Translation

Translation of the vRNA occurs at the surface of the ER and results in the synthesis of a highly membrane-associated polyprotein product. This polyprotein is further processed by host and cellular proteases co- and post-translationally through an ordered process that presumably dictates the ER topology of the mature viral proteins. Following the entry of the virion into the target cell, the first round of translation must take place in order to produce all viral proteins (including the viral RNA polymerase), which are absent from the infectious virus particle. Hence, vRNA translation is a critical step for the initiation of vRNA synthesis and subsequent amplification. The flaviviral genome, like cellular messenger RNAs (mRNA), contains a cap structure at the 5′ end which enables translation through canonical cap-dependent translation initiation (Garcia-Blanco et al., 2016). The addition of the cap is mediated by NS5 protein’s methyltransferase activity in combination with the nucleotide triphosphatase activity of NS3. NS3 removes a phosphate from the 5′ terminus of the vRNA and NS5 catalyzes the addition of guanosine monophosphate (GMP) as well as the methylation of both this guanine on N-7 and the ribose-2′ OH of the first adenosine to form a type 1 5′ cap structure (m^7^GpppAm2) (Egloff et al., 2002; Ray et al., 2006; Klema et al., 2016; Wang et al., 2018). Remarkably, in contrast to cellular mRNAs, vRNA lacks a 3′ poly-A tail. The poly-A tail is typically important for stability and to stimulate translation initiation of cellular mRNAs due to its strong association with poly-A-binding protein (PABP), which interacts with the cap-binding complex eIF4F and mediates the circularization of the mRNA. Despite the lack of a poly-A tail, the 3′ end of DENV RNA can associates with PABP *in vitro* (Polacek et al., 2009). This interaction appears to be specific for A-rich sequences flanking the DB structures upstream the terminal 3′SL motif of the 3′UTR (Figure 1A). Moreover, in *in vitro* translation assays using cell extracts, treatment with Paip2, an inhibitor of PABP, repressed translation of a reporter mRNA containing the DENV2 5′UTR, the first 72 nt of capsid coding sequence and the 3′UTR in a dose-dependent manner. This suggests that PABP/3′UTR interaction mimics the role of mRNA poly-A tail and presumably stimulates translation initiation. In addition, the 3′SL structure also modulates DENV translation. However, since this motif is functional *in vitro* within mRNAs containing either an internal ribosome entry site (IRES) or a non-functional cap, it was proposed that the 3′SL independently influences translation after cap binding by the small 40S ribosomal subunit (Holden and Harris, 2004).

Another stem loop structure in 5′UTR named “capsid-coding region hairpin” (cHP) is also implicated in flaviviral translation (Clyde and Harris, 2006). Mutations in the cHP sequence which abrogate its secondary structure decreased initiation from the first AUG codon resulting in the production of shorter capsid protein products expressed from reporter RNAs in human hepatoma Hep3B and mosquito C6/36 cells. This highlights that cHP is important for initiating translation from the correct start codon and for generation of a functional capsid protein. Moreover, viral translation also relies on two pseudoknot motifs within the 3′UTR called 5′ψ and 3′ψ. They involve two identical dumbbells structures termed 5′-DB and 3′-DB (or DB1 and DB2, respectively) which are both flanked by A-rich regions. Their formation is promoted by the presence of “conserved sequences” RCS2 and CS2 in 5′-DB and 3′-DB, as well as their respective terminal loops which both contain five nucleotide sequences named TL1 and TL2 (Figure 1A). TL1 and TL2 are complementary to pentanucleotide sequences PK1 and PK2 downstream of each DB. TL1/PK2 and TL2/PK1 tertiary interactions constitute the 5′ψ and 3′ψ pseudoknots, respectively (Olsthoorn and Bol, 2001; Manzano et al., 2011). Similar structures have also been reported for other flaviviruses such as JEV and YFV (Olsthoorn and Bol, 2001). Manzano and colleagues have also reported that the TL1 and TL2 are important for flavivirus translation in BHK-21 cells, but their respective contributions to translation appear unequal (Manzano et al., 2011). Indeed the deletion of TL2 impaired translation only modestly while disruption of TL1 had no effect. However, the deletion of both sequences resulted in a more severe phenotype strongly suggesting that TL1 and TL2 act synergistically to enhance translation from the DENV vRNA. A similar phenotype was observed when TL1 and TL2 were swapped. Importantly, mutations abrogating TL/PK complementarity impeded translation, which returned to wild-type levels by mutations that restored base pairing, highlighting the importance of these tertiary interactions. However, in contrast to TL1 and TL2, PK1 and PK2 are not absolutely necessary for translation suggesting that alternative TL receptors within the vRNA might exist. For instance, when the PK1 sequence is mutated, TL2 might interact with the top loop of 3′-SL. Taken together, these observations highlight that this core RNA region is crucial for the regulation of efficient viral translation.

In addition to canonical initiation of translation, cap-independent mechanisms of translation have also been described for DENV. Indeed, DENV can achieve vRNA translation and wild-type production of infectious viral particles when cap-dependent translation is inhibited by treating the cells with drugs that impair the phosphoinositol-3 kinase (PI3K) pathway. Moreover, expression knockdown of eIF4E (a component of the eiF4F cap-binding complex) in hamster BHK-21 or monkey Vero cells led to a 60% decrease in total cellular protein synthesis, whereas DENV NS5 protein levels decreased by just 10% (Edgil et al., 2006). This data suggests that DENV translation initiation can also occur in a cap-independent manner. DENV cap-independent translation appears to be regulated by both 5′ and 3′UTRs. Nonetheless, no IRES has been identified for flaviviruses in contrast to virus from other genus within the *Flaviviridae* such as hepatitis C virus (HCV) (Perard et al., 2013).

Using polysome profiling, Roth and coworkers have demonstrated that all tested flaviruses (namely all DENV serotypes, pathogenic WNV, historical and contemporary ZIKV strains) induce a general shut-off of host cell translation early following infection in human hepatocarcinoma Huh7 cells (Roth et al., 2017). This DENV-induced translation inhibition occurs at the initiation step. Interestingly, another group has recently shown that DENV infection in Huh7 cells negatively regulates the translation of host mRNAs that are associated with the ER without impacting the synthesis cytosolic proteins (Reid et al., 2018). In contrast, translation of vRNA appears to be unaffected by this global inhibition strongly supporting that flaviviruses specifically divert host protein synthesis for the benefit of viral translation and/or other steps of the life cycle. Importantly, cellular stresses such as infection or oxidative stress induce perturbations in cell translation (Anderson and Kedersha, 2008). More specifically, such stresses can induce translational arrest associated with polysome disassembly, and a concomitant appearance of stress granules (SG). SGs are cytoplasmic granules composed of untranslated mRNAs and the translation initiation machinery comprising proteins of the 48S preinitiation complex including eIF3, eIF4A, eIF4G, PABP1 and small ribosomal subunits (Anderson and Kedersha, 2008). In most of the cases, the formation of SGs requires the phosphorylation of eIF2α by protein kinase R (PKR) or PKR-like endoplasmic reticulum kinase (PERK). In its phosphorylated form, eIF2α inhibits global protein translation by reducing levels of the eIF2α-GTP-tRNA^Met^ ternary complex, which is absolutely required for translation initiation. Surprisingly, it appears that DENV-mediated repression of translation initiation is not functionally linked to PKR, infection-associated eIF2α phosphorylation or SG induction. These observations are consistent with several studies that have reported that DENV, ZIKV and WNV infection inhibits the formation of SGs, especially when cells are under oxidative stress following treatment with the SG inducer sodium arsenite (Emara and Brinton, 2007; Amorim et al., 2017; Roth et al., 2017). In such conditions, reductions in the number of formed SGs and a decrease of phospho-eIF2α levels are observed in infected cells. This inhibition seems to be specific to eIF2α-specific SGs since ZIKV infection did not impact of the formation of SGs upon pateamine A or sodium selenite treatments which do not require prior eIF2α phosphorylation and are devoid of the SG marker TIA-1-related protein (TIAR) (Amorim et al., 2017). Interestingly, eIF2α-specific SG components T cell internal antigen-1 (TIA-1) and TIAR, which are known to induce translational silencing (Anderson and Kedersha, 2008) are diverted by flaviviruses to regulate replication (Li et al., 2002; Emara and Brinton, 2007). Indeed, in infected BHK-21 cells, these factors colocalize with viral proteins and dsRNA within the replication complex. Such relocalization has also been observed in TBEV-infected cells and it was proposed that these host factors inhibit the translation of the TBEV vRNA (Albornoz et al., 2014). Overall, these studies support the idea that flaviviruses manipulate host cell gene expression at the translational level to favor viral protein production and generate a cellular state which is favorable to replication.

## vRNA Replication

### Overview of the vRNA Synthesis Process

vRNA replication is the core step leading to virus amplification and consists of *de novo* RNA synthesis (i.e., without initiation from a preexisting primer). Within RFs (see below), it generates a pool of neosynthesized vRNA molecules that are subsequently used for the formation of new replication complexes, for translation-driven production of viral proteins and for packaging into assembling virus particles. vRNA replication relies on the RNA-dependent RNA polymerase (RdRp) activity of flaviviral NS5 protein, through an asymmetric process. vRNA synthesis is initiated by binding of NS5 to a secondary structure located at the 5′ terminus of the genome called “stem loop A” (SLA) which is critical for the initiation of vRNA synthesis (Filomatori et al., 2006). NS5 synthesizes first one molecule of negative-strand intermediate RNA using the positive-strand vRNA as a template. Subsequently, new copies of vRNA are made from this negative strand RNA, with a higher proportion of positive-strand vRNAs produced (You and Padmanabhan, 1999; Guyatt et al., 2001). NS5 requires both 5′ and 3′UTRs to initiate negative-strand RNA synthesis (Filomatori et al., 2006; Hodge et al., 2016). For the synthesis of this antigenome, the vRNA must adopt a circularized panhandle-shaped structure formed through long range interactions between the 5′ and 3′UTRs. This conformation allows to position the 5′ and 3′UTR in close proximity and to transfer SLA-bound NS5 from the 5′UTR to the 3′ stem loop (3′SL) located at the terminus of the 3′UTR. More specifically, NS5 interacts with the top loop of the 3′SL which is highly conserved across the *Flavivirus* genus (Hodge et al., 2016). This configuration of NS5 enables the initiation of negative-strand synthesis using a pppAG dinucleotide as a primer. Following antigenome synthesis, NS5 can polymerize many copies of the positive-strand vRNA from the negative-strand intermediate. NS5 is optimized to specifically use the pppAG dinucleotide as a primer. As a result, 3′ CU and 5′ AG (3′ CU in the antigenome) termini of vRNA are strictly conserved among flaviviruses (Selisko et al., 2012). The reasons why the flavivirus RNA synthesis is asymmetric in favor of the positive-strand RNA (i.e., vRNA) are still unclear. However, it has been proposed that in the double-stranded RNA state (vRNA/antigenome hybrid), vRNA SLA-bound NS5 molecules would be directly transferred from neosynthetized vRNA to the 3′ end of the negative-strand (instead of vRNA 3′SL) to directly reinitiate positive-strand synthesis (Garcia-Blanco et al., 2016). This is consistent with a JEV study that indicated a greater affinity of NS5 for the 3′ end of the negative-strand RNA than for vRNA 3′UTR (Kim et al., 2007). This model of asymmetric viral RNA replication supports the idea that the negative-strand would not be free in the cell but rather annealed with both template and/or neosynthesized vRNA molecules.

Other RNA secondary structures in the 3′UTR have also been reported to influence replication. For instance, in addition to their role in translation (as discussed above), the 5′ψ and 3′ψ tertiary structures in the DENV vRNA regulate RNA synthesis (Olsthoorn and Bol, 2001; Manzano et al., 2011). Indeed, mutations in PK or TL sequences disrupting the pseudoknots result in a decrease of viral replication. Similarly to the translation phenotype, the contributions of TL1 and TL2 to viral RNA replication are not equivalent. However, in contrast to what has been observed for translation, restoration of base pairing between the TL and PK sequences does not rescue the replication defects caused by individual mutations. This pinpoints that there are differences between the roles of 5′ψ and 3′ψ in translation and replication, in line with the idea that changes in the conformation of the vRNA modulate the different steps of the viral life cycle.

It is believed that NS3 helicase assists vRNA synthesis presumably through direct interactions with NS5 (Johansson et al., 2001; Takahashi et al., 2012). Although this helicase activity is absolutely required for flavivirus life cycle, it remains unclear which exact step of vRNA synthesis it regulates in infected cells. Nevertheless, based on *in vitro* analyses, several models have been proposed. NS3 helicase activity is most likely involved in vRNA synthesis from the negative strand. According to the model described above in which vRNA synthesis is initiated from dsRNA, NS3 would be required to unwind this molecule and displace the original vRNA molecule in favor of the nascent genome. Moreover, it cannot be excluded that NS3 also contributes to the synthesis of the antigenome by unwinding secondary and tertiary structures in vRNA. Finally, NS3 might unwind the RNA duplex so that neosynthesized positive-strands can be translated or packaged into assembling viral particles.

### Genome Circularization

As discussed above, the cyclization of the vRNA is critical for the initiation of genome replication through the recruitment of NS5 to SLA in the 5′UTR. vRNA shows a high structural plasticity with ample evidence to suggest that several sub-domains act as riboswitches to regulate the different steps of the viral life cycle, including initiation of RNA synthesis (Figure 1B). Firstly, DENV vRNA can adopt different conformations during infection, and switching from circular to linear conformations modulates negative and positive-strand RNA synthesis (Villordo et al., 2010). This structural plasticity relies on the highly structured 5′ and 3′UTRs, with the presence of a variety of stem loop structures termed “cyclization sequences” (or elements). Through sequence complementarity, they contribute to long range RNA-RNA interactions and hence, promote the circularization of the flaviviral genome. Notably, evidence of individual molecules of circularized DENV vRNA has been provided *in vitro* using atomic force microscopy (Alvarez et al., 2005). One of the main cyclization elements involved in this process are the “conserved sequences” (CS). They are constituted of 8 or more nucleotides located in the 5′ region of the capsid-coding sequence and in the 3′UTR. CSs were first identified in WNV and have been demonstrated to be essential for flaviviral replication in BHK-21 cells (Khromykh et al., 2001a). However, Alvarez and colleagues later demonstrated that the base pairing between the 5′ and 3′ CS alone was necessary, but not sufficient for vRNA circularization *in vitro*. They demonstrated that other cyclization motifs contribute to this long range RNA-RNA interaction (Alvarez et al., 2005). Indeed, the “upstream AUG regions” (5′ UAR) located before the polyprotein start codon in “stem loop B” (SLB) interacts with the 3′ UAR which overlaps with the “small hairpin” (sHP) in the highly conserved 3′SL at the terminus of 3′UTR (Figure 1). The “downstream AUG region” (DAR) long range interaction is also an important determinant of genome circularization. Of note, DAR are not very conserved among flaviviruses and while DENV show only one DAR interaction, WNV and YFV seem to rather possess a bipartite element (named DAR1 and DAR2) (Friebe et al., 2011; Brinton and Basu, 2015). In the case of DENV, a region of 6 nucleotides identified in the 5′ region of the genome (5′DAR) is involved in DENV replication and possibly also RNA cyclization. The 3′DAR sequence mapping to the 5′ stem of sHP is complementary to the 5′DAR (Friebe and Harris, 2010; Villordo et al., 2010). Consistently, sHP has been demonstrated to be implicated in long-range RNA–RNA interactions, with a major contribution from the UAR-containing stem. Moreover, DENV harboring mutations in the stem of this structure replicates less efficiently than wild-type virus. However, while mutations in the 3′ DAR sequence impact viral replication, its role in genome circularization through interactions with 5′ DAR is less clear. In fact, 3′ DAR mutations would rather influence the stability and/or the formation of sHP and this may explain their overall impact on viral genome replication. Friebe and Harris have hypothesized that the 5′-3′ DAR interaction might not be needed to make the 3′UAR accessible to the 5′ UAR. Instead, they propose that the UAR, CS, DAR and cHP sequences constitute a functional unit essential for the circularization of vRNA (Friebe and Harris, 2010). In addition to its role in start codon selection during translation (see above), the cHP structure has also been shown to be important for DENV and WNV vRNA synthesis in a sequence-independent manner. How cHP influences vRNA synthesis remains to be determined; however, one possibility is that, within this functional unit, it contributes to the formation and/or stabilization of the vRNA panhandle structure (Clyde et al., 2008).

A sequence present downstream of the 5′ CS element in the capsid-coding sequence called “downstream CS” (dCS) is important for flavivirus replication and this sequence impacts genome circularization by modulating the topology of the 5′ end. In line with this, changes in the dCS sequence composition affects the formation of the 5′-3′ long range RNA-RNA interactions (Friebe et al., 2012). Moreover, an RNA motif termed “downstream of 5′ CS pseudoknot” (DCS-PK) also enhances replication in BHK-21 cells by regulating circularization (Liu et al., 2013). This tertiary interaction localizes to the capsid-coding region and appears to be constituted by a three-stem pseudoknot structure. Disruption of the DCS-PK structure hinders the ability of the 5′ RNA to bind 3′ RNA, while the rescue of DCS-PK structure recovered the formation of this 5′-3′ interaction. It was proposed that both dCS and DCS-PK contribute to the function of the cyclization unit containing 5′UAR, 5′CS, 5′DAR, and cHP. In this model, the DCS-PK sequence might help this unit to adopt specific conformations which favor genome circularization.

A structure present downstream SLA in the 5′UTR has been recently identified as an important riboswitch, which controls the equilibrium between NS5 recruitment to SLA and the circularization of the vRNA. This motif named “5′ UAR-flanking stem” (UFS) is a U-rich region located in SLB that promotes the formation of a conserved duplex RNA (Liu et al., 2016). The conformation of UFS is critical for the recruitment of NS5 to SLA and the SLA-dependent initiation of RNA synthesis. Indeed, mutations disrupting UFS result in a decrease in NS5 binding to the 5′UTR *in vitro* and consequently of replication. In contrast, increasing the stability of the UFS does impede vRNA circularization. If UFS is too stable, this could result in a “locked-up” conformation of the UAR sequence which is known to be implicated in long range RNA-RNA interactions. Consistently, the circularization of vRNA induced the melting of the UFS structure resulting in a decrease in affinity of NS5 for the 5′ end of the vRNA. These data support a model in which the UFS functions as a riboswitch during RNA replication, which dictates vRNA circularization and NS5 recruitment. Following the binding of NS5 to SLA, the circularization of the vRNA would induce a disruption of the UFS structure, leading to a decrease in the affinity between NS5 and the 5′UTR. This would favor NS5 transfer to the 3′UTR, hence properly positioning the polymerase for negative-strand RNA synthesis.

### Viral Replication Factories

A striking feature of flaviviral infections is the appearance of organelle-like membranous replication factories (RF) resulting from severe alterations of ER membranes. The detailed tridimensional architecture of RFs from several flaviviruses has been reconstructed using electron tomography (Welsch et al., 2009; Gillespie et al., 2010; Miorin et al., 2013; Junjhon et al., 2014; Bily et al., 2015; Cortese et al., 2017). RFs are constituted of several sub-structures namely vesicle packets (VP), convoluted membranes (CM), and virus bags (VB) which are morphologically different and can be found within the same ER network.

**FIGURE 2:**
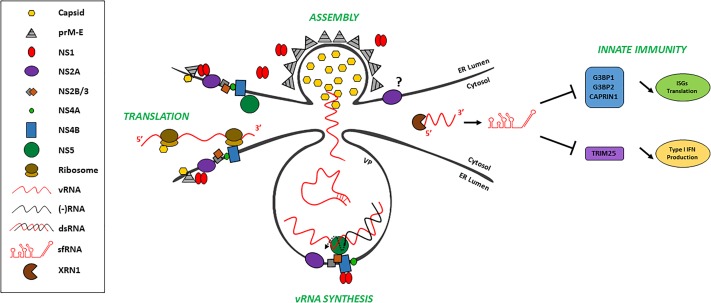
A model of the different fates of vRNA. vRNA is replicated by NS5 within the vesicle packets (VP) with the assistance of other nonstructural proteins. Using vRNA, the positive strand (red), as a template, the synthesis of the negative strand (black) by NS5 is depicted. Subsequently, several new copies of vRNA are produced from the dsRNA replication intermediate (not shown). Translation presumably occurs outside of VPs since ribosomes can be visualized adjacent to these structures. vRNA is proposed to exit VPs through a pore to be directly encapsidated and enveloped into juxtaposed ER budding structures. Finally, vRNA is partially degraded by cellular XRN1 which generate sfRNA. sfRNA regulates several host responses including innate immunity at the levels of signal transduction and ISG translation.

Vesicle packets are spherical vesicles which are induced by invaginations of the ER (Figure 2). They show similar morphology in both mosquito and mammalian cells suggesting that their biogenesis relies on evolutionary conserved host machineries and pathways. In mammalian cells, their diameter is approximately 90 nm and they are connected to the cytoplasm by a 10 nm-wide pore. Interestingly, it was shown that in the case of WNV and TBEV, vesicles within the same ER cisternae are also connected to each other by pore-like openings suggesting that they exchange material (Gillespie et al., 2010; Offerdahl et al., 2012; Miorin et al., 2013). The determinants of both types of pores are completely unknown. Immunogold labeling combined with electron microscopy has revealed that VPs contain dsRNA, the replication intermediate as well as several viral non-structural proteins absolutely required for replication such as NS5, NS3, NS1, NS4A and NS4B (Welsch et al., 2009; Miorin et al., 2013; Junjhon et al., 2014). Hence, it is strongly believed that vRNA synthesis takes place in this compartment. Nevertheless, it remains unclear if VPs are absolutely required for replication or if other ER sub-compartments can support replication. Furthermore, it is also thought that VPs constitute an environment favorable to vRNA synthesis. Indeed, they may play a role in protecting the vRNA from degradation by nucleases or recognition by cytosolic sensors of RNA, dampening the activation of antiviral signaling pathways. Finally, VPs would allow the concentration of metabolites, as well as cellular and viral factors required for efficient vRNA synthesis. However, these models remain to be experimentally validated.

Convoluted membranes are large reticular structures enriched in NS2B/3, NS4A and NS4B that are induced by membrane curvature and morphologically resemble tight accumulations of smooth ER membranes (Westaway et al., 1997; Miller et al., 2007; Welsch et al., 2009; Chatel-Chaix et al., 2016). The exact role of CMs is not well understood; however, they have been recently proposed to modulate cellular processes such as innate immunity or inter-organellar communication in order to create a proviral cytoplasmic environment rather than to directly regulate vRNA synthesis *per se* (Chatel-Chaix et al., 2016).

Newly assembled virions accumulate in regular arrays into VBs which are dilated ER cisternae (Welsch et al., 2009; Cortese et al., 2017). VPs and VBs may be found in close proximity within the same ER network, which contains ribosomes on its cytosolic side. This suggests that RFs provide a platform for the transfer of viral genomes between replication complexes, ribosomes and assembling virus particles. Moreover, this confers a spatial segregation of the different vRNA-containing complexes allowing the coordination of vRNA translation, replication and encapsidation in both space and time.

### *Trans* Co-factors Involved in vRNA Replication

All flaviviral NS viral proteins are absolutely required for vRNA synthesis (Apte-Sengupta et al., 2014; Selisko et al., 2014); yet, only NS5 and NS3 possess enzymatic activities. The transmembrane proteins NS1, NS4A and NS4B are believed to be implicated in the formation of RFs. Notably, when transiently expressed alone, DENV and WNV NS4A are able to induce to some extent the formation of CMs in Huh7 and Vero cells, respectively (Roosendaal et al., 2006; Miller et al., 2007). NS1 was shown to alter liposome membrane *in vitro* (Akey et al., 2014). Considering that all of the NS proteins physically and/or genetically interact, it is tempting to speculate that they synergistically act to coordinate the different steps of vRNA replication. For instance, the interaction between NS5 and NS3 seems to be important to functionally couple vRNA synthesis and dsRNA unwinding (Yu et al., 2013; Tay et al., 2015). Moreover, the DENV NS3 helicase domain associates with the cytosolic loop of the NS4B transmembrane protein (Umareddy et al., 2006; Chatel-Chaix et al., 2015; Zou et al., 2015a). NS4B mutants that lose the capacity to interact with NS3 are defective in replication suggesting that the integrity of this complex is critical for DENV life cycle. Interestingly, NS4B was shown to promote the dissociation of NS3 from single-stranded RNA (Umareddy et al., 2006) implying that it would indirectly stimulate the recruitment of the helicase toward newly formed replication intermediates and would promote their unwinding. Finally, NS4B homodimerizes and interacts with both NS1 and NS4A (Youn et al., 2012; Zou et al., 2014, 2015b; Chatel-Chaix et al., 2015; Li et al., 2015). This further supports that protein-protein interactions coordinate the activity of the replication complexes with a precise ER membrane topology within VPs.

Finally, numerous cellular RNA-binding proteins have been reported to interact with flaviviral vRNA and to modulate genome replication. Some examples are listed in Table 1. While vRNA binding motifs have been identified in some studies, the precise molecular mechanisms by which these proteins modulate the viral life cycle remain unclear in most cases. Some proteins show specificity for vRNA motifs. For example, TIA-1 and TIAR interact with negative-strand RNA 3′ SL of WNV and the knockout of these proteins decreases viral titers implicating these interactions in efficient viral replication (Li et al., 2002). In contrast, DDX6 and NF90 modulate DENV replication presumably through their interaction with the DB and 3′ SL structures in the 3′UTR of the vRNA, respectively (Gomila et al., 2011; Ward et al., 2011). Finally, the isoform p45 of host protein AUF1 (also named hnRNP D) was reported to positively regulate the replication of WNV, DENV and ZIKV in Huh7 cells by promoting vRNA circularization. Indeed, AUF1 destabilizes SLB and the 3′ SL thereby exposing the UAR circularization elements (Friedrich et al., 2014, 2018) (Figure 1B). This illustrates that host factors are able to impact viral genome plasticity and to regulate important riboswitches in the flaviviral vRNA.

**Table 1:** Host RNA-binding proteins involved in flavivirus life cycle.

Host factor	Virus	Role	Regulated step(s)	vRNA binding site	Reference
CSDE1	DENV	Proviral	Translation/replication?	UTR	Phillips et al., 2016
DDX3	JEV	Proviral	Translation	5′UTR + 3′UTR	Li et al., 2014
DDX5	JEV	Proviral	Translation/replication?	3′ UTR	Li C. et al., 2013
DDX6	DENV	Proviral	?	3′UTR (5′and 3′ DB)	Ward et al., 2011
eEF1α	DENV/WNV	proviral	Replication	DENV 3′UTR (between 3′CS and 3′ end)/WNV 3′ SL	Blackwell and Brinton, 1997; De Nova-Ocampo et al., 2002; Davis et al., 2007
ERI3	DENV/YFV	Proviral	Replication	3′UTR (DB)	Ward et al., 2016
FBP1	JEV	Antiviral	Translation	5′UTR + 3′UTR	Chien et al., 2011
hnRNP A2/B1	DENV/JEV	Proviral	?	DENV 3′UTR/JEV 5′ end of (-) RNA	Paranjape and Harris, 2007; Katoh et al., 2011
hnRNP C1/C2	DENV	Proviral	Replication? (not translation)	?	Dechtawewat et al., 2015; Phillips et al., 2016
hnRNP D/AUF-1	ZIKV/DENV/WNV	Proviral	Replication	5′UTR (SLB) + 3′UTR (3′SL)	Friedrich et al., 2014; Friedrich et al., 2018
hnRNP G/RBMX	DENV	Proviral	Translation/replication?	?	Viktorovskaya et al., 2016
La	JEV	Proviral	?	5′UTR + 3′UTR (3′ SL)	Vashist et al., 2009; Vashist et al., 2011
LSm1	DENV	Proviral	?	3′UTR	Dong et al., 2015
Musashi-1	ZIKV	Proviral	Translation/replication?	3′ UTR	Chavali et al., 2017
NF90	DENV	Proviral	Translation/ replication?	3′UTR (3′SL)	Gomila et al., 2011
p100	DENV	Proviral	Translation	3′UTR (between 3′ CS and 3′ end)	Lei et al., 2011
PABP	DENV	Proviral	Translation	3′UTR (3′SL)	Polacek et al., 2009; Phillips et al., 2016
PTB	DENV/JEV	Proviral (DENV)/antiviral (JEV)	Translation/replication	DENV 3′UTR/JEV 5′UTR+(-) RNA	De Nova-Ocampo et al., 2002; Agis-Juarez et al., 2009; Bhullar et al., 2014
QKI	DENV (DENV4 only)	Antiviral	Translation?	3′UTR	Liao et al., 2018
RPLP1/2	DENV/YFV/ZIKV	Proviral	Translation	?	Campos et al., 2017
TIA1/TIAR	WNV	Proviral	Replication	3′SL of (-) RNA	Li et al., 2002; Emara et al., 2008
YBX1	DENV	Antiviral/proviral?	Translation (antiviral)/virus production (proviral)	3′UTR (3′SL)	Paranjape and Harris, 2007; Phillips et al., 2016
ZAP	JEV	Antiviral	?	3′UTR (DB)	Chiu et al., 2018

*For each cellular co-factor, the virus(es), the known RNA-binding site(s) and the step(s) of its life cycle which is regulated are indicated. For simplification purposes, an indicated role in translation does not exclude an impact on vRNA stability. (-)RNA, Minus strand viral RNA*.

## vRNA Packaging

During virus assembly, the vRNA genome must be encapsidated into neosynthetized viral particles. This step of the flavivirus life cycle which is a prerequisite for full infectivity is one of the least characterized and understood at the molecular level. Our understanding of the vRNA packaging process remains limited due to the lack of identification of (presumably short-lived) assembly intermediates. Moreover, comprehensive studies about the intracellular distribution of the different vRNA populations are lacking.

The vRNA encapsidation process must face several “challenges” that intuitively, have to be tightly regulated. First, this process must be specific. Only vRNA is encapsidated while cellular RNA and viral negative-strand intermediate RNA must be excluded from the capsid. Second, the stoichiometry of the viral genome inside the virion (i.e., vRNA copy number per virion) is important for optimal infectivity (Kuhn et al., 2002; Byk and Gamarnik, 2016). The highly basic viral capsid protein binds with high affinity to the negatively charged vRNA in what is presumed to be a rather non-specific manner through electrostatic interactions (Pong et al., 2011; Byk and Gamarnik, 2016). However, as capsid molecules far outnumber the copies of vRNA in the virion, vRNA packaging must be regulated to achieve optimal vRNA intraviral stoichiometry (presumably one genome copy per virion) and full infectivity. In contrast to several other viruses like HIV-1 (Comas-Garcia et al., 2016), no *bona fide* RNA packaging signal has been identified for members of the *Flavivirus* genus. If packaging signal do exist, they are most likely to be located within the highly structured untranslated regions. Indeed, in the case of the related *Hepacivirus* HCV, the 3′UTR was shown to be important for RNA *trans*-encapsidation while mechanistic details are still being characterized (Shi et al., 2016). Identifying putative flaviviral packaging signals remains challenging because, if located in the UTRs, they are likely to overlap with motifs important for translation and vRNA synthesis. Hence, mutation of these putative motifs may also potentially affect viral replication (and indirectly downstream virus assembly), making it difficult to functionally segregate replication from vRNA packaging. Nevertheless, one study has identified a *cis*-acting RNA motif that influences virus assembly. Using a silent mutagenesis approach, Groat-Carmona and colleagues demonstrated that the conserved DENV “capsid-coding region 1” (CCR1) influences the production of infectious particles in both insect C6/36 and mammalian BHK-21 cells without affecting vRNA stability, translation and synthesis (Groat-Carmona et al., 2012). Importantly, DENV replication as well as dissemination from the mid-gut to the salivary glands in the mosquito vector relied on the integrity of the CCR1 structure, highlighting the importance of this RNA motif *in vivo*. Since CCR1 mutations resulted in a drastic reduction in infectious titers without affecting the levels of extracellular vRNA, a contribution of CCR1 in vRNA packaging was ruled out by the authors and its exact role in virus assembly is still unknown. Nevertheless, a putative reduction of vRNA packaging may have been masked by the presence of non-encapsidated newly synthesized vRNA in the cell supernatants, which could have been released from the cell within exosomes in a viral assembly independent manner. Hence, a possible role of CCR1 in vRNA packaging should likely be re-evaluated. As discussed above (see vRNA replication), the structural dynamics of the vRNA itself allow it to orchestrate the different steps of vRNA replication including vRNA circularization, NS5 binding and RNA synthesis. Considering that some vRNA domains can function as riboswitches, it is tantalizing to speculate that conformational changes in vRNA secondary and tertiary structures drive vRNA transfer from replication complexes in VPs to assembling virions. Moreover, the methylation status of the vRNA might contribute determining its fate. Indeed, it was recently shown that DENV, WNV, YFV, ZIKV and HCV vRNAs are N^6^-methylated on adenosines by the host methyltransferases METTL3 and METTL14 in infected cells (Gokhale et al., 2016; Lichinchi et al., 2016). Very interestingly, N^6^A-methylated ZIKV vRNA is associated with cellular YTHDF proteins that inhibit infectious particle production (Lichinchi et al., 2016). In the case of HCV, the same inhibition is observed and it correlates with the redistribution of YTHDF proteins to lipid droplets (the virus assembly site) while this did not influence the vRNA replication process. Thus, this strongly suggests that N^6^A methylation specifically regulates virus assembly (Gokhale et al., 2016). Based on these results and the possible conservation across the *Flaviviridae* family, one might hypothesize that only vRNA molecules that are not N^6^A-methylated are packaged into assembling viruses. In addition, it is reasonable to consider that the methylation of vRNA influences its folding and hence, its functions during the different steps of the viral life cycle. Such hypotheses will likely be challenged in future studies.

Although no vRNA packaging signal has been identified, it is well established that *trans*-encapsidation is possible for flaviviruses. Indeed, when structural proteins are expressed in *trans*, they form virus-like particles that can encapsidate sub-genomic replicons, i.e., replication-competent genomes that express only NS proteins (Khromykh et al., 1998; Ansarah-Sobrinho et al., 2008; Qing et al., 2010; Suzuki et al., 2014; Scaturro et al., 2015). The resulting *trans*-complemented particles are infectious and are able to undergo a single round of infection. Interestingly, the structural proteins are able to encapsidate genomes from other flaviviruses (Yoshii et al., 2008; Shustov and Frolov, 2010; Suzuki et al., 2014). This strongly suggests that the *cis* RNA and *trans* protein determinants of the vRNA packaging process are conserved across the *Flavivirus* genus. However, it remains elusive how the flaviviral genome is specifically selected for encapsidation. Like HCV core protein, flaviviral C protein accumulation on lipid droplets is important for the generation of infectious virus particles (Miyanari et al., 2007; Samsa et al., 2009; Carvalho et al., 2012; Martins et al., 2012; Iglesias et al., 2015). However, this pool of structural proteins may represent a storage compartment for assembly competent capsid rather than the actual site of genome selection and particle assembly. Interestingly, early studies on YFV and Murray Valley encephalitis virus (MVEV), another flavivirus, have highlighted that polyprotein processing and virus morphogenesis are functionally linked (Lobigs, 1993; Lee et al., 2000; Lobigs and Lee, 2004; Lobigs et al., 2010). Indeed, uncoupling these two processes by introducing mutations altering the processing kinetics of the signal peptide between capsid and prM, critically impaired nucleocapsid envelopment and the production of infectious viral particles. Most strikingly, several independent ultrastructural studies on DENV and ZIKV based on 3D reconstruction of replication factories revealed structures budding into the ER and juxtaposed to the pore of the VPs, the presumed site of vRNA replication (see above and Figure 2) (Welsch et al., 2009; Junjhon et al., 2014; Cortese et al., 2017). This pore was observed in 90% of DENV VPs and is homogenous in size (diameter of ∼10 nm) (Welsch et al., 2009). In the case of WNV, the RNA inside the VP is aligned with the pore (Gillespie et al., 2010). However, nothing is known about its morphogenesis and dynamics, and the viroporin activity of VP-associated NS2A, NS2B and NS4B transmembrane proteins might participate to this process (Chang et al., 1999; Leon-Juarez et al., 2016; Shrivastava et al., 2017). In addition, the juxtaposed budding structures have the size of assembled virions and contain an electron dense core which may correspond to a vRNA-containing capsid. Based on this observation, it is tempting to speculate that the vRNA replication and packaging processes are coordinated in time and space. The newly synthesized positive-strand genome molecule would exit the VP through the pore and be directly encapsidated into budding virions, hence conferring the selectivity of genome encapsidation. Such a coordinated model implies that the replication process and/or the presence of VPs would be required for vRNA encapsidation and envelopment by the ER membrane. This is further supported by early studies showing that replication is required for virus production in BHK-21 cells (Khromykh et al., 2001b). Indeed, DNA-launched WNV replication-deficient genomes fail to generate extracellular viral particles despite the presence of vRNA and structural proteins. It should be noted that replication is not required for the formation of sub-viral particles (i.e., devoid of the viral genome and non-infectious) whose budding can occur upon expression of prM/E alone (Schalich et al., 1996; Wang et al., 2009). Thus, it would be interesting to analyze the cellular RNA content of sub-viral particles since it is not known if they contain non-specifically enveloped cellular RNA or if they are free of nucleic acids. Importantly, it remains unclear how budding structures are physically juxtaposed to VPs and whether this event is absolutely required for the production of fully infectious virus. In addition to its critical function in replication, ZIKV and DENV NS1 were recently demonstrated to be important for both virus assembly and release (Scaturro et al., 2015; Yang et al., 2017). Consistently, ultrastructural studies have demonstrated that a fraction of DENV NS1 is associated with virions. Interestingly, mutants of the NS1 β-ladder domain lost their ability to indirectly associate with capsid while their interaction with glycoproteins E and prM was maintained (Scaturro et al., 2015). This suggests that NS1 might assist the specific envelopment of capsid/vRNA complexes in structures budding into the ER (Figure 2). Additionally, the expression level of WNV NS1’, an alternative larger form of NS1 resulting from a translational frameshift was shown to influence the specific infectivity in *trans*-complementation experiments in BHK-21 cells (Winkelmann et al., 2011). In addition, NS2A also has an influence on both RNA replication and viral particle production (Liu et al., 2003; Leung et al., 2008; Vossmann et al., 2015; Wu et al., 2015; Xie et al., 2015; Yang et al., 2017). Finally, NS3 has specific functions during particle assembly independently from its enzymatic functions. Indeed the W349A mutation in YFV NS3 impacted infectious particle production while vRNA replication and the release of sub-viral particles remained unaffected (Patkar and Kuhn, 2008). Very interestingly, DENV NS3 helicase domain was shown to possess an RNA annealing activity *in vitro* (Gebhard et al., 2012), implying that it can influence the conformation of vRNA in infected cells. This suggests that through specific interactions with the vRNA (Swarbrick et al., 2017), NS3 might promote the exposure of a putative packaging motif and directly regulate genome encapsidation and/or capsid envelopment. This regulation might also involve a contribution of assembling virions since WNV capsid protein, similarly to NS3, possesses an RNA chaperoning activity *in vitro* (Ivanyi-Nagy and Darlix, 2012). Taken together, all these findings support the idea that viral proteins bring together replication and assembly complexes to orchestrate an efficient and selective vRNA encapsidation process.

Host factors may also play a role in the tight regulation of vRNA packaging during virus assembly. Several cellular RNA-binding proteins have been reported to associate with the flaviviral genome mostly through the UTRs, and to be important for the viral life cycle (Table 1). In most studies, the authors did not identify the exact step controlled by their candidate protein or may not have considered vRNA packaging in the analyses. Interestingly, the RNA-binding protein DDX56 appears to be important for the production of infectious WNV particles, but not for vRNA replication, strongly suggesting that it acts during vRNA selection for encapsidation (Xu et al., 2011; Xu and Hobman, 2012; Reid and Hobman, 2017). Nevertheless, while virions released from DDX56 depleted cells contained less encapsidated vRNA, a DDX56-vRNA interaction remains to be demonstrated.

Several of the identified flaviviral replication co-factors such as YBX1, hnRNP K, DDX6 or DDX3 were reported to also associate with the genome of HCV (Ariumi et al., 2007; Paranjape and Harris, 2007; Jangra et al., 2010; Chatel-Chaix et al., 2011; Chahar et al., 2013; Chatel-Chaix et al., 2013; Li et al., 2014; Brunetti et al., 2015; Poenisch et al., 2015; Phillips et al., 2016). Those host factors are components of the same ribonucleoprotein complex (RNP) (Vashist et al., 2012; Chatel-Chaix et al., 2013; Upadhyay et al., 2013) and some of them have been reported to regulate the equilibrium between HCV RNA replication and the production of infectious viral particles suggesting that they control the transfer of vRNA from replication to assembly complexes (Chatel-Chaix et al., 2011; Chatel-Chaix et al., 2013). Whether the viral co-opting of this host RNP is conserved across the *Flaviviridae* family will have to be evaluated in the future. Nonetheless, it is likely that flaviviruses, as obligatory intracellular parasites, hijack the function of several host RNA-binding proteins during vRNA encapsidation. One can envisage that such co-opting would influence or be modulated by the various 3D structures and modifications of the vRNA. Interestingly, several of *Flaviviridae* vRNA-binding proteins, such as hnRNP C, hnRNP A2/B1 and RBMX (see Table 1) were showed to have enhanced affinity for N^6^A methylated RNAs whose local conformation is changed by this modification (Alarcon et al., 2015; Liu et al., 2015, 2017). This suggests a functional link between vRNA modifications, riboswitches and riboproteomic profiles. Thus, integration of all currently known models will likely help to provide a clearer understanding of how flaviviruses control genome selection for encapsidation.

## Flaviviral RNA and Innate Immunity

### vRNA and Pattern Recognition Receptors

During viral entry or RNA amplification, flaviviral RNA can be sensed as foreign RNA by the cell and trigger antiviral innate immunity in mammalian cells. This first line of defense involves RNA sensors that, once activated, trigger a signaling cascade leading to the production of interferons (IFN) and interferon-stimulated genes (ISG). ISGs are antiviral effectors that in some cases, specifically target vRNA, may be secreted as proinflammatory cytokines or generate an overall antiviral state to impede virus replication (Adachi et al., 1998). Pattern recognition receptors (PRR) such as Toll-like receptor 3 (TLR3), retinoic acid-inducible gene I (RIG-I) as well as melanoma differentiation-associated protein 5 (MDA5) are expert sensors of highly structured viral RNAs or dsRNA, and consequently, are implicated in antiflaviviral host responses (Loo et al., 2008; Nasirudeen et al., 2011; Sprokholt et al., 2017).

TLR3 is a member of the Toll-like receptor family and plays a crucial role in activation of the immune response by recognition of dsRNA in endosomes, presumably during viral entry (Leifer and Medvedev, 2016; Gao and Li, 2017). TLR3 recognizes DENV RNA in infected cells and its overexpression or stimulation reduces viral replication (Tsai et al., 2009; Liang et al., 2011). In a pathological context, TLR3 knockout mice are more susceptible to lethal WNV infection (Daffis et al., 2008).

RIG-I belongs to the RIG-I-like receptor (RLR) family and possesses a dsRNA helicase activity. It is a cytosolic PRR that targets specifically dsRNA and the 5′ tri/diphosphate moiety of short structured uncapped RNAs (Yoneyama et al., 2004; Pichlmair et al., 2006; Takahasi et al., 2008; Goubau et al., 2014). It also has been shown to have affinity for the polyuridine tract of HCV 3′UTR (Schnell et al., 2012). Such sequences are very unusual in cellular RNAs and hence, constitute foreign signatures. Interestingly, treatment of cells or mice with U-rich 5′ppp-based RIG-I agonists protect from infection with variety of viruses (Chiang et al., 2015). MDA5 is another RLR family member related to the RIG-I protein. MDA5 targets long viral dsRNAs and activates the same innate antiviral response as RIG-I (Schlee, 2013). Once activated by RNA recognition, RIG-I and MDA5 interact with “mitochondrial antiviral-signaling protein” (MAVS) at the surface of mitochondria through their CARD domains. This interaction results in a signaling cascade to the nucleus via transcription factors NF-κB and IRF3. This ultimately leads to the induction of type I IFN, proinflammatory cytokines and ISGs expression (Gack and Diamond, 2016).

More recently, it has been shown that the cyclic GMP-AMP synthase (cGAS)/stimulator of IFN genes (STING) pathway, which normally senses DNA virus infection and mitochondrial DNA damage, is also activated upon RNA virus infection (including DENV and WNV) and induces type I IFN production (Schoggins et al., 2014). The vRNA sensing mechanism is still not well understood but DENV-induced mitochondrial damage may be involved in this process (Aguirre et al., 2017; Sun et al., 2017). Moreover, the relevance of this pathway to flavivirus infection is further highlighted by several evidence that DENV NS2B and NS3 are able to counteract the functions of cGAS and STING, respectively (Aguirre et al., 2012, 2017; Yu et al., 2012).

### Flaviviral RNA-Based Evasion From the Innate Immune System

From a virus-host co-evolution viewpoint, the mammalian innate immune response has evolved to counteract viral infection. Of course, this also implies an adaptation from the pathogens in order to evade innate immunity to the benefit of replication. To this end, several interference mechanisms involving flaviviral proteins have been described over the last decade. Indeed, these viruses can dampen the antiviral signaling pathways by inhibiting for instance, cGAS, STING, RIG-I, MAVS, TBK1 and STAT1/2 functions through interactions with NS5, NS3, NS2B or NS4B (Chatel-Chaix et al., 2016; Gack and Diamond, 2016; Aguirre et al., 2017; Miorin et al., 2017). In addition, the viral genome itself and its degradation by-products also contribute to the efficient evasion of innate immunity. Firstly, as mentioned above, vRNA most likely replicates within VPs, constituting a confined environment providing limited access to cytosolic vRNA sensors. Hence, from an ultrastructural perspective, it is tempting to speculate that VPs “hide” vRNA and the dsRNA replication intermediate from the innate immune detection machinery. Nevertheless, such models remain to be addressed specifically.

#### vRNA Methylation and Innate Immunity

Interestingly, several studies have shown that vRNA modifications may confer the vRNA with the ability to be marked as “self” and evade recognition by host sensors of foreign RNA. Indeed, in addition to vRNA capping, NS5 also possesses a 2′-*O*-methyltransferase activity (Bradrick, 2017). 2′-*O*-methylation is an RNA modification on the first and second nucleotides of the mRNA cap structures in which the ribose is methylated at the 2′-OH position by cellular nucleoside 2′-O-methyltransferases (MTase) contributing to form cap 1 (m^7^GpppNm) or cap 2 (m^7^GpppNmNm) structures (Furuichi and Shatkin, 2000). Hence, through NS5-mediated 2′-*O* methylation of its cap, the flaviviral vRNA mimics cellular mRNAs. Moreover, DENV and WNV NS5 proteins were demonstrated to also perform internal RNA methylation on vRNAs that lack the 5′ cap structure (Dong et al., 2012). In this case, these modifications occur specifically at the 2′-OH position of adenosine residues.

By mimicking cellular mRNA, modified vRNAs appear to evade the host immune response during infection. Indeed, DENV 2′ O-MTase deficient viruses are severely attenuated and do not properly spread in cell lines that possess a functional IFN response system (like lung carcinoma A549 cells) (Schmid et al., 2015; Chang et al., 2016). In the case of WNV, a virus expressing the NS5 E218A mutant which lacks the 2′-O MTase activity is attenuated in primary cells and mice with a strongly reduced pathogenicity including the complete loss of virus-induced lethality (Daffis et al., 2010). Importantly, the pathogenicity of this mutant virus *in vivo* was restored in mice harboring a deficiency in type I interferon signaling. This strongly supports the idea that 2′-*O*-methylation is crucial to evade the type I IFN-dependent antiviral response. Notably, mutant and wild-type viruses induce comparable levels of IFNs suggesting that WNV vRNA sensing by RLR or TLR3 is not involved in this evasion strategy. Importantly, this methylation-dependent antiviral effect was attributed to IFN-induced proteins with tetratricopeptide repeats (IFIT). More specifically, replication and pathogenicity of WNV E218A mutant virus was rescued and comparable to wild-type virus in *Ifit1* knockout mice demonstrating the key role of this ISG in antiviral immunity (Daffis et al., 2010). As compared to other IFITs, IFIT1 recognizes with high specificity RNAs lacking a 2′-O methylated cap. This results in the sequestration of these RNAs from translation initiation factors and consequently, in the inhibition of their translation (Habjan et al., 2013; Kimura et al., 2013; Kumar et al., 2014). Nevertheless, *Ifit1* deficiency did not rescue the replication WNV E218A in brain endothelial cells in contrast to other cell types of the central nervous system (Szretter et al., 2012). Consistently, overexpression of IFIT1 in 293-DC-SIGN cells only partially inhibited the replication of DENV2 2′-O MTase mutant (Zust et al., 2013). This highlights that the 2-*O*-methylation of vRNA allows evasion from innate immunity and relies on both IFIT1-dependent and independent mechanisms according to the cell type.

Interestingly, the role of virus-mediated 2′-*O*-methylation as a countermeasure against innate immunity has also been recognized in mouse and human coronaviruses. In this case, 2′-O MTase-deficient viruses induced a stronger type I IFN response resulting in attenuation of viral replication (Zust et al., 2011; Schmid et al., 2015). However, coronaviral replication was restored upon suppression of type I IFN receptor (IFNAR) or cytosolic RNA sensor MDA5 expression suggesting that 2′-O methylation of the coronavirus RNA directly evades early RNA sensing by MDA5. Whether the same strategy is also employed by flaviviruses (other than WNV) remains to be elucidated. Interestingly, a recent study has demonstrated that a DENV E216A 2′-O MTase-deficient mutant induced an early innate immune response after just a few hours of infection, consistent with a putative detection of unmethylated vRNA by RLRs such as MDA5 or RIG-I (Chang et al., 2016).

Since 2′-O-methylation is important for optimal viral replication and also has structural similarities among flaviviruses, it was proposed that 2′-O MTase-deficient viruses could be exploited as attenuated vaccines. Indeed, several groups have engineered attenuated DENV or JEV that lack 2′-*O*-methylation activity and are thus more sensitive to IFN inhibition than parental viruses (Li S.H. et al., 2013; Zust et al., 2013). Robust humoral and cellular immune responses protecting against both viruses were obtained after inoculation of mice with these attenuated viruses. In the case of DENV, protection was also achieved in rhesus macaques after a single administration of the vaccine candidate (Zust et al., 2013). These results pinpoint the potential success of such attenuated vaccine-based approach, which may be efficacious against a wide range of flaviviruses.

#### The Action of sfRNA Against Innate Immunity

During the infection, the accumulation of viral genome generates several by-products which do not encode any viral proteins. Three classes of non-coding RNAs have been described to date: viral small RNAs (vsRNAs) (Hamilton and Baulcombe, 1999), defective interfering genomes (DIGs) (Li and Brinton, 2001; Pesko et al., 2012; Juarez-Martinez et al., 2013) and most relevant to this review, the subgenomic flavivirus RNA (sfRNA) (Urosevic et al., 1997; Pijlman et al., 2008). DIGs and vsRNAs are not well described yet and remain to be characterized in detail. In contrast, the sfRNA has been comprehensively investigated during the last decade, especially with regards to its role in modulating host biological processes.

Produced by all tested members of the *Flavivirus* genus, sfRNA is a highly structured 0.3–0.7 kb-long non-coding RNA and apparently is the most abundant viral RNA species in the infected cell (Pijlman et al., 2008; Bidet et al., 2014; Manokaran et al., 2015; Akiyama et al., 2016; Donald et al., 2016; Bidet et al., 2017). It is well established that sfRNA is produced by an incomplete 5′-3′ degradation of the viral genome by the cellular XRN1/Pacman exonuclease. During RNA degradation, XRN1 is stalled at the 3′UTR extremity, more precisely at stem-loops/pseudoknots of the highly variable region upstream the DB structures causing the accumulation of different species of sfRNA (Pijlman et al., 2008; Funk et al., 2010; Silva et al., 2010; Chapman et al., 2014a,b; Akiyama et al., 2016). While XRN1 is required for sfRNA production, sfRNA is also able to sequester this cellular protein and to inhibit its endogenous functions (Silva et al., 2010; Moon et al., 2012; Chapman et al., 2014a). In 293T cells, this results in the accumulation of uncapped cellular mRNAs in the cytosol (Moon et al., 2012). However, the consequences of such inhibition are still unclear and remain to be further deciphered.

Despite its high levels in the cytosol, sfRNA does not appear to play a direct role in replication since mutations impairing sfRNA production do not affect vRNA synthesis in WNV-, YFV-, and DENV-infected cells (Funk et al., 2010; Schuessler et al., 2012; Szretter et al., 2012). Rather, sfRNA contributes to viral cytopathicity both *in cellulo* and *in vivo* partly by interfering with innate immune responses. For instance, in the case of WNV and DENV infection, mutations inhibiting sfRNA production lead to a decrease in the viral replication in cells that possess functional type I IFN responses, supporting the idea that sfRNA aids in evasion of the IFN response (Schuessler et al., 2012; Bidet et al., 2014, 2017). Moreover, a recent study shows that DENV sfRNA negatively impacts IFN induction through the inhibition of TRIM25, an E3 ubiquitin ligase required for RIG-I activation (Manokaran et al., 2015). Indeed, the interaction of sfRNA with TRIM25 in a sequence-dependent manner prevents its deubiquitylation. As a result, the decrease in IFN induction is consistent with an impairment in TRIM25-mediated polyubiquitylation and subsequent activation of RIG-I. Consistent with a conservation of this evasion strategy among flaviviruses, it was shown that JEV sfRNA overexpression in infected A549 cells inhibits IRF3 phosphorylation and its nuclear translocation that are required for type I IFN transcription (Chang et al., 2013). Finally, the RLR-dependent IFN induction pathway was also inhibited upon ZIKV sfRNA overexpression in stimulated cells (Donald et al., 2016).

Downstream of IFN production and signaling, the sfRNA also plays a role in ISG expression at the post-transcriptional level. Indeed, host RNA-binding proteins G3BP1, G3PB2 and CAPRIN, which are involved in ISG translation are inhibited by their association with DENV sfRNA in Huh7 cells (Bidet et al., 2014). G3BP proteins are core components of SGs (Anderson and Kedersha, 2006) whose formation and functions are modulated by flavivirus infection, as discussed above. Hence, it is tempting to speculate that, through the hijacking of SG components, flaviviruses remodel the host proteome by positively or negatively regulating the expression of pro- and anti-viral host proteins, respectively.

Interestingly, several studies have shown that sfRNA also plays an important role in flaviviral life cycle and dissemination in infected insects (Schnettler et al., 2012; Moon et al., 2015; Pompon et al., 2017). The determinants of the viral genome governing the abundance of sfRNA appear to be the same in insect and mammalian cells; however, in mosquitos, the sfRNA causes disruption of the innate immune response in salivary glands by inhibiting the Toll receptor pathway (Pompon et al., 2017). Furthermore, sfRNA downregulates the RNA interference (RNAi) machinery, the main mediator of innate immunity in insects (Schnettler et al., 2012; Moon et al., 2015). Mutations in DENV and WNV decreasing the production of sfRNA showed an impairment in RNAi suppression (Moon et al., 2015). This appears to be mediated by the association of sfRNA with Dicer and Ago2, two essential proteins of the RNAi machinery. Taken together, these data suggest that the sfRNA crucially contributes at multiple levels to viral evasion from innate immunity in both arthropod and mammalian hosts.

## sfRNA-Mediated Modulation of Pathogenesis

In addition to its roles in innate immune evasion, the sfRNA was shown to be important for WNV and DENV pathogenicity. WNV or DENV genomes harboring mutations that disrupt the formation of full-length sfRNA produced much smaller plaques in cell culture (Pijlman et al., 2008; Liu et al., 2014). Consistently, drastic decreases in overall cell death and apoptosis were observed. These phenotypes were rescued by the expression of the sfRNA in *trans*. However, sfRNA overexpression alone did not induce any cell death implying that its action requires flavivirus replication. Importantly, viral translation, vRNA synthesis and particle production were not significantly affected by the loss of sfRNA expression. Overall, this strongly suggests that sfRNA is not essential for flavivirus replication but rather modulates cytopathic effects in addition to innate immunity. Interestingly, DENV sfRNA-mutated viruses were unable to inhibit Bcl2 and the AKT/PI3K pro-survival pathways suggesting that flavivirus-induced cytopathic effects rely on the modulation of these signaling cascades (Liu et al., 2014). Most importantly, mice infected with full length sfRNA-deficient WNV all survive in contrast to the usual 100% mortality rate with wild-type WNV (Pijlman et al., 2008). This did not correlate with defects in virus dissemination in the brain and spleen confirming that sfRNA is crucial for pathogenicity *in vivo* without directly regulating viral replication. In stark contrast, overexpression of JEV sfRNA decreased virus-induced apoptosis in infected A549 cells (Chang et al., 2013). While it is clear that sfRNA is crucial for WNV pathogenicity and that all tested flaviviruses produce sfRNA (Pijlman et al., 2008; Bidet et al., 2014, 2017; Manokaran et al., 2015; Akiyama et al., 2016; Donald et al., 2016), their respective contributions to pathogenesis remain to be addressed. Finally, how sfRNA modulates the flavivirus-induced cytopathic effects at the molecular level is completely unknown. It will be interesting to determine if sfRNA acts at the gene expression level or rather post-translationally through direct interactions with factors involved in cell survival and/or cell death.

## Open Questions and Conclusion

The tremendous work on flaviviruses during the last decade highlights the complexity of the molecular mechanisms governing the fate and functions of the vRNA. This includes dynamic RNA secondary and tertiary structures, RNA modifications such as 2′-*O* and N^6^A methylation, the formation of functional vRNA sub-products (like sfRNA) and the participation of viral proteins as well as host RNA-binding proteins. This intricate network is most likely hosted within viral RFs. Future studies will be needed to explore how all these regulated processes are interconnected to generate a precise integrated model of vRNA metabolism. For instance, does vRNA methylation on specific nucleotides impact vRNA tertiary structure formation, cyclization and/or affinity for host RNA-binding proteins, and vice-versa? Do these structural changes impact the efficiency of vRNA packaging into assembling virions? Importantly, when compared with HCV RFs, little is known about how flaviviruses regulate the morphogenesis of VPs that are very homogenous in size and shape. The same applies to the formation and maintenance of the VP pore that is believed to play a pivotal role in the transfer of vRNA from replication complexes to assembling particles. How is it functionally coordinated with budding viruses? Is this a dynamic structure oscillating between open and closed states? What is its viral and cellular protein composition? Finally, these considerations should ideally always take into account that flaviviruses infect both insects and mammals. Indeed, subtle differences between hosts in the life cycle (especially with regards to host factor dependency) may be observed and of great interest.

Overall, this review highlights how flaviviruses have evolved to confer upon a single RNA species and one viral polyprotein product all the information required for optimal infection in both insect and mammalian hosts. More generally, all of these open questions regarding the vRNA perfectly illustrate the importance of flaviviruses as an exquisite model to study spatio-temporal control of RNA metabolism. Finally, a precise understanding of the dynamic control of vRNA in the flavivirus life cycle will hopefully identify potential therapeutic targets for the development of antivirals, ideally with a broad pan-flaviviral spectrum.

## Author Contributions

CM and WF contributed equally to this work. CM and WF wrote the manuscript and made the figures. LC-C edited the final version of the manuscript.

## Conflict of Interest Statement

The authors declare that the research was conducted in the absence of any commercial or financial relationships that could be construed as a potential conflict of interest.
